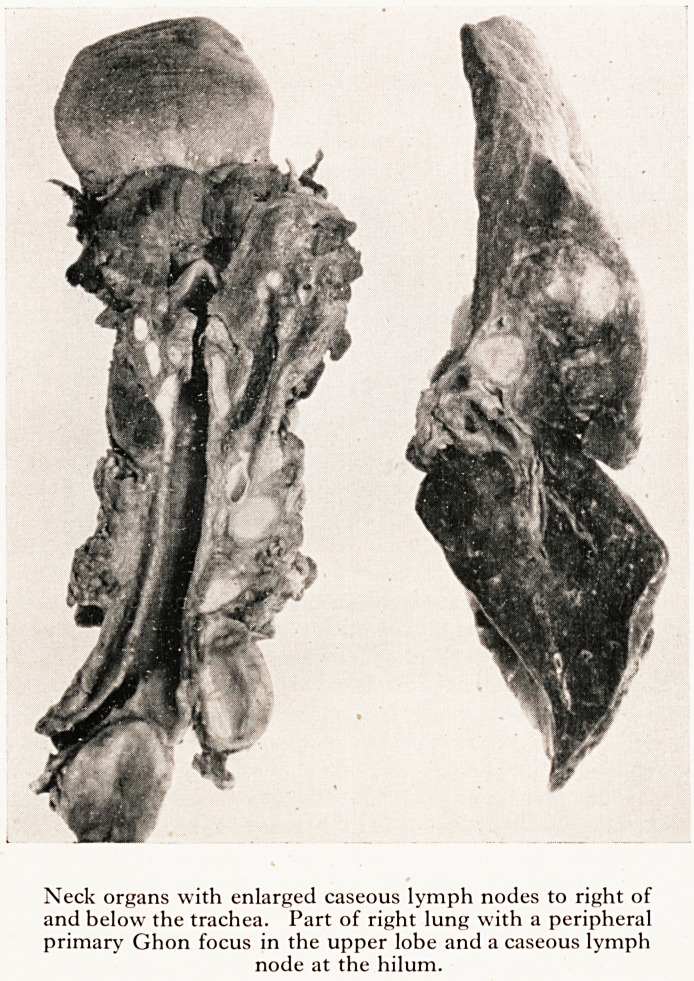# Mongolism, Hypertrophic Pyloric Stenosis Treated with "Skopyl" and Death from Miliary Tuberculosis and Meningitis
*A case report at a Clinico-Pathological Conference held on 23rd February, 1954, in the Department of Pathology, University of Bristol.


**Published:** 1954-07

**Authors:** 


### MONGOLISM, HYPERTROPHIC PYLORIC STENOSIS TREATED WITH "SKOPYL" AND DEATH FROM MILIARY TUBERCULOSIS AND MENINGITIS*

*Dr. Beryl Corner:* This baby was born in hospital and was recognized as a mongol shortly after birth. His progress during the first fortnight was reasonably satisfactory. When he was three weeks old he started vomiting and was readmitted aged five weeks. On readmission the child was dehydrated and there was clear clinical evidence of pyloric stenosis. Medical treatment was started using the drug "Skopyl", which is prepared in Sweden and has been tried out on a research series of cases of infantile pyloric stenosis in Bristol. This case was included in the research group. The baby was perhaps a little slower to respond than some, and a little vomiting continued for the first few days of treatment, whereas most babies stop vomiting at once. Subsequently all vomiting ceased and he made good progress. The baby was a severe example of mongolism with flabby musculature and poor appetite, but good progress was eventually made and he went out of hospital gaining weight, taking feeds well and in every way, clinically, a healthy baby. Children in this series were seen regularly up to two years after treatment to ensure that the condition had been cured rather than symptomatically relieved; therefore this child was seen at intervals and X-rays demonstrated satisfactory functional progress. I saw him up to June, 1953, when he was about eighteen months old; he was doing quite well despite the fact that the mother was not very intelligent and the baby often appeared looking dirty. There were no symptoms and when he was X-rayed in July, stomach emptying time and the pyloric canal were both apparently normal. I had made an appointment to see him in November, but he did not keep the appointment and the Health Visitor reported that the baby had bronchitis. He was admitted to hospital as an emergency in a moribund condition at the end of November. The admission diagnosis was acute bronchopneumonia; râles were heard all over his chest; he was in a moribund condition and died shortly afterwards.

*Professor Hewer:* Would Dr. Corner tell us something about "Skopyl" and its use?

*Dr. Corner:* "Skopyl" is a trade name for methylscopolamine nitrate. The important point about this drug is that the methylation of the scopolamine makes its peripheral effect on the alimentary tract very much intensified as compared with the central action. This drug was used experimentally in Scandinavia, not originally for pyloric stenosis, but for relief of various types of colic, and was found to be more effective than methyl atropine nitrate in its action on the gut so that it was eventually used for congenital hypertrophic pyloric stenosis in infancy. I have treated 110 cases with this particular drug since 1949, and have not submitted one of these babies to operation since the first three months of the research trial. Clinically, the effect is quite dramatic. The majority of babies stop vomiting within three days and about 35 per cent. never vomit again after the drug has been started. I have seen no toxic effects from it in the dosage which we give, which has been a matter for experimentation. Treatment is given regularly until the child is about sixteen weeks old, reducing the dose gradually towards the end of the period of treatment, since at sixteen weeks, without any treatment, pyloric stenosis symptoms usually begin to lessen. "Skopyl" has proved extremely satisfactory in this clinical trial; most of the cases have been

*A case report at a Clinico-Pathological Conference held on 23rd February, 1954, in the Department of Pathology, University of Bristol.

followed up clinically and radiologically and this is the only one who has died, and therefore it is the only one in which we have had a chance to examine the pyloric canal after treatment.

In reply to a question by Professor Bruce Perry, Dr. Corner said the heart was normal.

*Dr. B. E. McConnell:* Is " Skopyl " used in the treatment of pertussis, as is eumydrin ?

*Dr. Corner:* Not so far as I know: it is in very short supply.

*Dr. G. R. Airth:* The initial findings in December 1951 were typical of developed hypertrophic pyloric stenosis. In July 1952, the pyloric canal was still long, narrow and irregular but gastric emptying commenced immediately. In November 1952, the canal was shorter but still of irregular diameter. In July 1953, the canal showed a well-defined mucosal pattern throughout and the stomach emptied rapidly.

These findings indicate successful medical treatment of the spastic obstruction, but it must be realized that they give no information concerning the regression or persistence of the pyloric tumour.

*Dr. H. Urich:* The post-mortem findings can best be discussed under three headings: (1) mongolism, (2) congenital hypertrophic pyloric stenosis, and (3) the terminal infection.

(1) The body was rather small for a child of two, and weighed 8,400 gm. It showed typical mongoloid stigmata: a small, round skull, eyes set widely apart with epicanthic folds, a small nose with a flattened bridge, short square hands with simian creases (on the left palm only) and short curved little fingers. The heart showed no congenital malformation. I could not detect any gross abnormalities in the brain other than those due to the terminal infection.

(2) The stomach was of normal size and shape. It showed no dilatation or hypertrophy in the body of the stomach, and the mucosal pattern was normal. The pyloric canal was elongated. Its length was 2 cm.; its wall was grossly hypertrophied and 3 mm. thick. The lumen was adequate and the mucosal pattern normal. (Plate XVI.)

Histological examination showed that the elongation and thickening of the wall of the pyloric canal was due entirely to gross hypertrophy of the circular muscle, typical of congenital hypertrophic pyloric stenosis.

It therefore appears that in spite of a complete functional recovery the underlying structural abnormality remained largely unaffected by treatment.

(3) The right lung showed a round, whitish, caseating nodule 1 cm. in diameter, in the upper lobe. The corresponding peribronchial lymph nodes as well as those in the mediastinum were grossly enlarged and showed extensive caseation. The appearances were typical of a primary tuberculous complex. (Plate XVII.)

The remaining parts of the lung showed numerous small whitish or greyish nodules, about 1 mm. in diameter. Similar lesions were found in the liver and kidneys, as well as in the spleen where they were somewhat larger and showed caseation in the centre. The lesions had the appearance of miliary tubercles.

The brain showed some flattening of the convolutions. The base of the brain was covered with thick gelatinous exudate which spread symmetrically around the brain stem, extended into the lateral fissures and over the roof of the fourth ventricle. Minute whitish granules were visible in some parts of the exudate, particularly along the course of the vessels. Cut sections of the brain showed internal hydrocephalus with dilatation of all four ventricles. The appearances were those of tuberculous meningitis.

Histological examination confirmed the tuberculous nature of all the lesions. Sections of lung, liver and spleen showed typical miliary tubercles consisting of lymphocytes, epithelioid cells and giant cells of the Langhans type. Larger foci showed extensive caseation. The meninges showed extensive infiltration with lymphocytes and epithelioid cells, extending into the adventitial spaces around the cortical vessels. The arteries in the affected meninges showed arteritis with

PLATE XVI

Part of stomach and duodenum showing persistent hypertrophy of muscle in the wall of the elongated pylorus.

PLATE XVII

Neck organs with enlarged caseous lymph nodes to right of and below the trachea. Part of right lung with a peripheral primary Ghon focus in the upper lobe and a caseous lymph node at the hilum.

proliferation of the intima and almost complete occlusion of some of the smaller vessels.

Sections stained by Ziehl-Neelsen's method showed scanty acid-fast bacilli in the lesions.

To sum up, the case was one of mongolism with congenital hypertrophic pyloric stenosis, from which he made a good functional recovery, and death from generalized miliary tuberculosis and tuberculous meningitis.

*Dr. Corner:* Following this post-mortem report a search for contact with an open case of tuberculosis revealed that a friend of the family, who had been visiting them almost daily during August 1953, was subsequently proved to have severe open phthisis from which she had since died.

*Dr. R. L. Bishton:* Did the brain show any sign of mongolism?

*Dr. Urich:* As I have not been able to find a clear description of the changes in the brain in mongolism, I would rather not answer that question.

*Professor Hewer:* Dr. Norman is an expert on the pathology of mongolism: will he kindly tell us about it?

*Dr. R. M. Norman:* Dr. Urich is right when he says that the text-books give little consistent guidance as to the neuropathology of mongolism. As a matter of fact, the naked-eye appearances of the brain in mongolism are much more characteristic of the condition than are the microscopical. The small round mongol skull owes its characteristic shape to a post-natal retardation of growth of the cartilaginous base and this abnormal shape imposes itself upon the brain. It is unusual to find the brain weight much above 1,100 gm., and in the majority the weight is nearer the thousand mark. Viewed from the side the contour of the occipital lobe rises almost vertically from the pole and the frontal lobes are stunted so that the whole brain looks globular. A common feature is the narrow, poorly developed superior temporal gyrus which is present in more than half the cases. A less common but well established abnormality is a relatively small cerebellum and brain stem—a persistence of the state of affairs normally present at birth and another instance of the "foetalism" of the mongol. Most writers speak of the simple convolutional pattern in this condition. While this impression is usually given by superficial examination, one often sees on section that the larger gyri are mushroomed at the crowns and overlap smaller, hidden convolutions. It seems that there is a real crowding together of the gyri and at times one may even see an actual fibrous fusion at points in the outer layers of contiguous convolutions.

It is commonly stated that the nerve cells are grossly diminished in the mongol cortex and at first sight it seems a reasonable notion that such low-grade defectives should have fewer neurones than the normal. In point of fact it is quite difficult to demonstrate areas where an obvious reduction in nerve cells has occurred—at any rate in mongols who have survived infancy, and it is certainly true to say that such a finding is not a widespread or a constant feature. No doubt circulatory anomalies especially those associated with congenital heart disease may account for the patchy "areas of clearing" sometimes present and perhaps smaller factors underlie the mild fibrillary gliosis often found in the white matter. In general, however, cell counts made in corresponding cortical areas in mongol and normal brains reveal either no difference or even an increase in favour of the mongol. This is due to a patchy clumping together of relatively immature nerve cells. It may well be that the total number of nerve cells in the mongol brain equals the normal but that, corresponding to the small over-all size of the brain, these nerve cells are more crowded together, are less well developed individually and have, therefore, less opportunity for synaptic connection with each other.

One other feature of interest may be mentioned. It is well known that the mongol ages prematurely and this is reflected in the brains of some of those who reach the forties by the presence of senile plaques and the neurofibrillary change of Alzheimer.

## Figures and Tables

**Figure f1:**
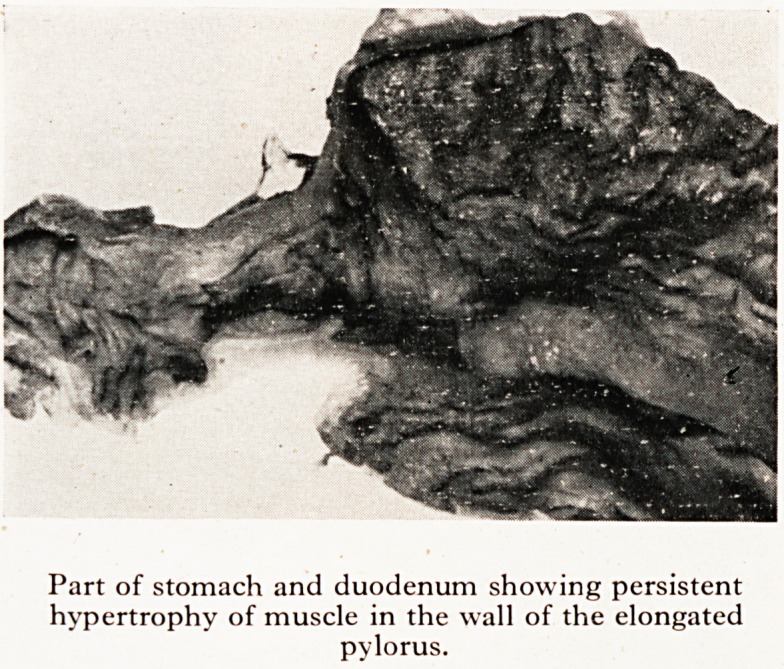


**Figure f2:**